# Neutralizing Antibodies against Lassa Virus Lineage I

**DOI:** 10.1128/mbio.01278-22

**Published:** 2022-06-22

**Authors:** Tierra K. Buck, Adrian S. Enriquez, Sharon L. Schendel, Michelle A. Zandonatti, Stephanie S. Harkins, Haoyang Li, Alex Moon-Walker, James E. Robinson, Luis M. Branco, Robert F. Garry, Erica Ollmann Saphire, Kathryn M. Hastie

**Affiliations:** a La Jolla Institute for Immunology, La Jolla, California, USA; b Department of Pediatrics, Tulane University School of Medicine, New Orleans, Louisiana, USA; c Zalgen Labs, LLC, Germantown, Maryland, USA; d Department of Microbiology and Immunology, Tulane University School of Medicine, New Orleans, Louisiana, USA; e Program in Virology, Harvard University, Boston, Massachusetts, USA; f Department of Molecular Microbiology, Washington University in Saint Louis, St. Louis, Missouri, USA; Stanford University; University of Pennsylvania

**Keywords:** Lassa virus, Lassa fever, antigenic variation, prefusion glycoprotein, cryo-EM, neutralizing antibodies, structure-based vaccine design, hemorrhagic fever virus, LAMP1, structure-guided immunogen, viral escape

## Abstract

Lassa virus (LASV) is the causative agent of the deadly Lassa fever (LF). Seven distinct LASV lineages circulate through western Africa, among which lineage I (LI), the first to be identified, is particularly resistant to antibody neutralization. Lineage I LASV evades neutralization by half of known antibodies in the GPC-A antibody competition group and all but one of the antibodies in the GPC-B competition group. Here, we solve two cryo-electron microscopy (cryo-EM) structures of LI GP in complex with a GPC-A and a GPC-B antibody. We used complementary structural and biochemical techniques to identify single-amino-acid substitutions in LI that are responsible for immune evasion by each antibody group. Further, we show that LI infection is more dependent on the endosomal receptor lysosome-associated membrane protein 1 (LAMP1) for viral entry relative to LIV. In the absence of LAMP1, LI requires a more acidic fusion pH to initiate membrane fusion with the host cell relative to LIV.

## INTRODUCTION

The Old World arenavirus Lassa virus (LASV) is the etiologic agent of the often fatal Lassa hemorrhagic fever (LF) (1). LASV is endemic to West Africa and exhibits a case fatality rate of greater than 50% in hospitalized patients and up to 90% in the third trimester of pregnancy (1, 2). LASV is carried by the common peridomestic rodent Mastomys natalensis and is transmitted to humans primarily through inhalation of aerosolized rodent excretions (3). Seven distinct LASV lineages (LI to LVII) are currently recognized and are arranged geographically according to the range of the host rodent populations (4, 5). Lineages V to VII were identified within the last 7 years (6–8). Lineage I (LI) of LASV was responsible for the first documented case of LF in 1969, when two missionary nurses were fatally infected and a third individual had severe illness (9). Although few LF cases linked to LI have since been reported, LI circulates in a conflict zone in northeastern Nigeria that lacks the hospitals and infrastructure needed to treat LF patients and conduct medical research (10). Thus, LI infections in this region are likely underreported.

LASV is an enveloped, negative-stranded RNA virus with an ambisense, bisegmented genome that encodes the following four proteins: the matrix protein (Z), viral polymerase (L), nucleoprotein (NP), and glycoprotein precursor (GPC) (11, 12). GPC is trafficked from the endoplasmic reticulum (ER) through the compartment, where it is heavily *N*-glycosylated and undergoes processing by the cellular proteases SPase and SKI-1/S1P to produce the mature, prefusion glycoprotein (GP) (13). Each GP monomer consists of the following three noncovalently associated subunits: the stable signal peptide (SSP), the receptor binding subunit (GP1), and the fusion machinery (GP2) (14, 15). Three GP monomers associate on the viral surface to form a trimer that drives viral attachment and cell entry (16).

Two host cell receptors, α-dystroglycan (αDG) and lysosome-associated membrane protein 1 (LAMP1), facilitate LASV viral entry. LASV GP binds a matriglycan sugar on the cell surface receptor αDG at alkaline pH, which triggers viral uptake via macropinocytosis (13, 17–19). In the endosome, GP undergoes a pH-driven receptor switch to the internal endosomal receptor, LAMP1 (20). Interaction between LASV GP and LAMP1 requires protonation of a histidine triad (H92, H93, and H230 in LIV) on GP1, which occurs when the endosomal pH drops below 6.0 (21, 22). In the absence of LAMP1, LASV infection can still occur, albeit with reduced efficiency (23).

LASV GP is the sole antigen on the viral surface and is the primary target of the adaptive immune response (24). Passive delivery of neutralizing monoclonal antibodies (MAbs) protects nonhuman primates (NHPs) from severe LF, even when administered at low doses and late in the disease course (25). The MAbs that are currently known to have neutralizing activity are categorized into the following four competition groups: GP1-A, GPC-A, GPC-B, and GPC-C (26). GP1-A MAbs require only the GP1 subunit for binding, while GPC-A, GPC-B, and GPC-C MAbs bind quaternary epitopes on the prefusion conformation of GP. GPC-A epitopes involve residues essential for LAMP1 binding and, for some, also involve residues in the GP2 fusion loop (27). GPC-B MAbs recognize a quaternary epitope bridging two neighboring monomers at the GP2 base of the trimer (28, 29) and comprise the majority of neutralizing LASV MAbs identified to date (26).

Currently, no vaccine or therapeutics are approved to prevent LASV infection or treat LF. The only existing treatment for patients with severe LF is ribavirin, a nucleoside analog that is effective only if administered during the early stages of infection (30). The wide genetic diversity of LASV introduces an additional challenge for vaccine development, since not all neutralizing antibodies are equally effective against the seven lineages (5, 31). Lineage I is particularly resistant to neutralization by GPC-A and GPC-B antibodies: many antibodies neutralize other lineages but fail to neutralize authentic LI LASV (28, 29). One GPC-A antibody is known to be pan-LASV and neutralize LI: 25.10C (26). The GPC-B antibody 18.5C, which does not naturally neutralize LI, can be engineered to do so by introduction of two Arg residues into complementarity-determining regions (CDRs) H2 and H3 (28, 29). The engineered antibody is termed 18.5C-M30, but no structure is yet available that explains how it acquired LI neutralization capacity.

Here, we present the first high-resolution structures of the prefusion GP (pfGP) ectodomain of LASV LI in complex with Fab fragments of GPC-A and GPC-B antibodies. Using complementary biochemical analyses, we examine the underlying mechanisms responsible for the ineffective neutralization of LI by these antibodies. Further, we show that LI-GP is more dependent on LAMP1 for cell entry relative to LIV-GP. These data shed light on naturally occurring substitutions in LASV GP that markedly reduce antibody efficacy in two of the dominant anti-LASV antibody competition groups. This information provides critical insights that will help inform the design of immunogens capable of eliciting neutralizing, pan-Lassa antibodies, which may also be resistant to viral escape.

## RESULTS

### Structural conservation between divergent lineages of LASV GP.

**FIG 1:**
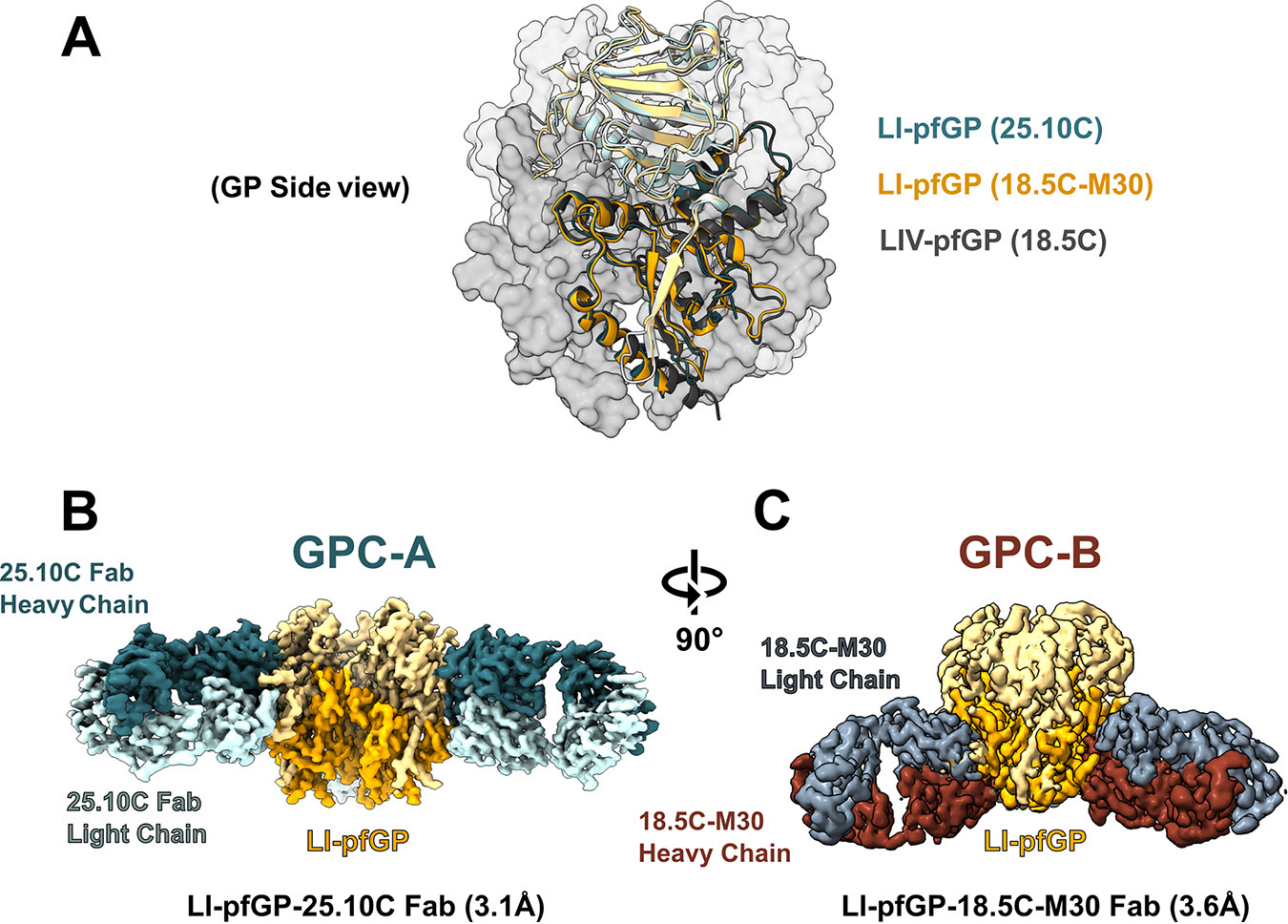
Structural characterization of the lineage I Lassa virus prefusion glycoprotein. (A) Superimposition of each of the LI-GP trimer structures, with the LIV-GP trimer (PDB accession number 7S8H) shown. GP structures were aligned by a single GP monomer from each structure, resulting in an average RMSD of 1.2 Å between LI and LIV. (B) Side-view of the LI-pfGP-25.10C cryo-EM reconstruction. GP1 and GP2 are colored in light and dark gold, respectively. The light- and heavy-chain domains of the 25.10C Fab are colored light and dark green, respectively. (C) Side-view of the LI-GP-18.5C-M30 cryo-EM reconstruction. GP1 and GP2 are colored in light and dark gold, respectively. The light- and heavy-chain domains of the 18.5C-M30 Fab are colored blue and maroon, respectively. The orientation of the LI-pfGP is rotated 90° from panel B to panel C.

We determined two structures of lineage I GP (LI-pfGP) as follows: a 3.1-Å cryo-electron microscopy (cryo-EM) structure of LI-pfGP in complex with GPC-A antibody 25.10C and a 3.6-Å cryo-EM structure of LI-pfGP in complex with GPC-B antibody 18.5C-M30 (Fig. 1; see also Fig. S1 to S3 and Tables S1 and S2 in the supplemental material).

10.1128/mbio.01278-22.1 FIG S1 Data processing and validation of LI-pfGP-25.10C. (A) Representative micrograph displaying LI-pfGP-25.10C Fab complexes. Scale bar, 20 nm. (B) Two-dimensional cryo-EM class averages of the LI-pfGP trimer in complex with 25.10C Fab (turquoise), depicting the 3:1 binding stoichiometry between LI-pfGP and 25.10C. (C) Fourier shell correlation (FSC) depicting the global resolution of the final LI-pfGP-25.10C cryo-EM reconstruction with 3-fold rotational symmetry applied. (D) Fourier shell correlation (FSC) between the sharpened cryo-EM reconstruction and atomic model with masked and unmasked FSC shown in blue and red, respectively. (E) Viewing direction distribution histogram depicting the diversity of particle projection views in the data set. (F) Three-dimensional representation of the viewing direction distribution of the particle projections used for the final cryo-EM reconstruction. Download FIG S1, TIF file, 2.5 MB. Copyright © 2022 Buck et al. 2022 Buck et al. https://creativecommons.org/licenses/by/4.0/ This content is distributed under the terms of the Creative Commons Attribution 4.0 International license.

10.1128/mbio.01278-22.8 TABLE S1 LI-pfGP in complex with Fab 25.10C. Download Table S1, DOCX file, 0.01 MB. Copyright © 2022 Buck et al. 2022 Buck et al. https://creativecommons.org/licenses/by/4.0/ This content is distributed under the terms of the Creative Commons Attribution 4.0 International license.

10.1128/mbio.01278-22.9 TABLE S2 LI-pfGP in complex with Fab 18.5C-M30. Download Table S2, DOCX file, 0.01 MB. Copyright © 2022 Buck et al. 2022 Buck et al. https://creativecommons.org/licenses/by/4.0/ This content is distributed under the terms of the Creative Commons Attribution 4.0 International license.

Overall, both LI GP structures share the same architecture as LIV GP (PDB accession number 7S8H), with an average root mean square deviation (RMSD) of 1.2 Å when aligned to a single GP monomer within the trimer (Fig. 1A). Three copies of both 25.10C and 18.5C-M30 Fab fragments bind a single LI GP trimer. As found with our recent structure of LIV bound to 25.10C (27), the heavy chain of 25.10C primarily contacts residues required for LAMP1 binding in GP1 (loop 225 to 235), while the light-chain anchors to the GP2 fusion loop (Fig. 1B). The GPC-B antibody 18.5C-M30, which is derived from GPC-B MAb 18.5C (28, 29), binds to a quaternary epitope spanning two adjacent monomers and bridging their GP1 to GP2 subunits (Fig. 1C). LI differs from other lineages at several key sites that could affect antibody activity, including position 95 in the GPC-A epitope and residues 62, 198, and 397, which are located in or near the GPC-B epitope. Here, we use the structures of LI-pfGP in complex with 25.10C and 18.5C-M30 to understand how these amino acid differences allow LI to evade neutralization by most known anti-LASV antibodies.

### LI Arg 95 decreases the efficacy of GPC-A antibody 36.1F.

**FIG 2:**
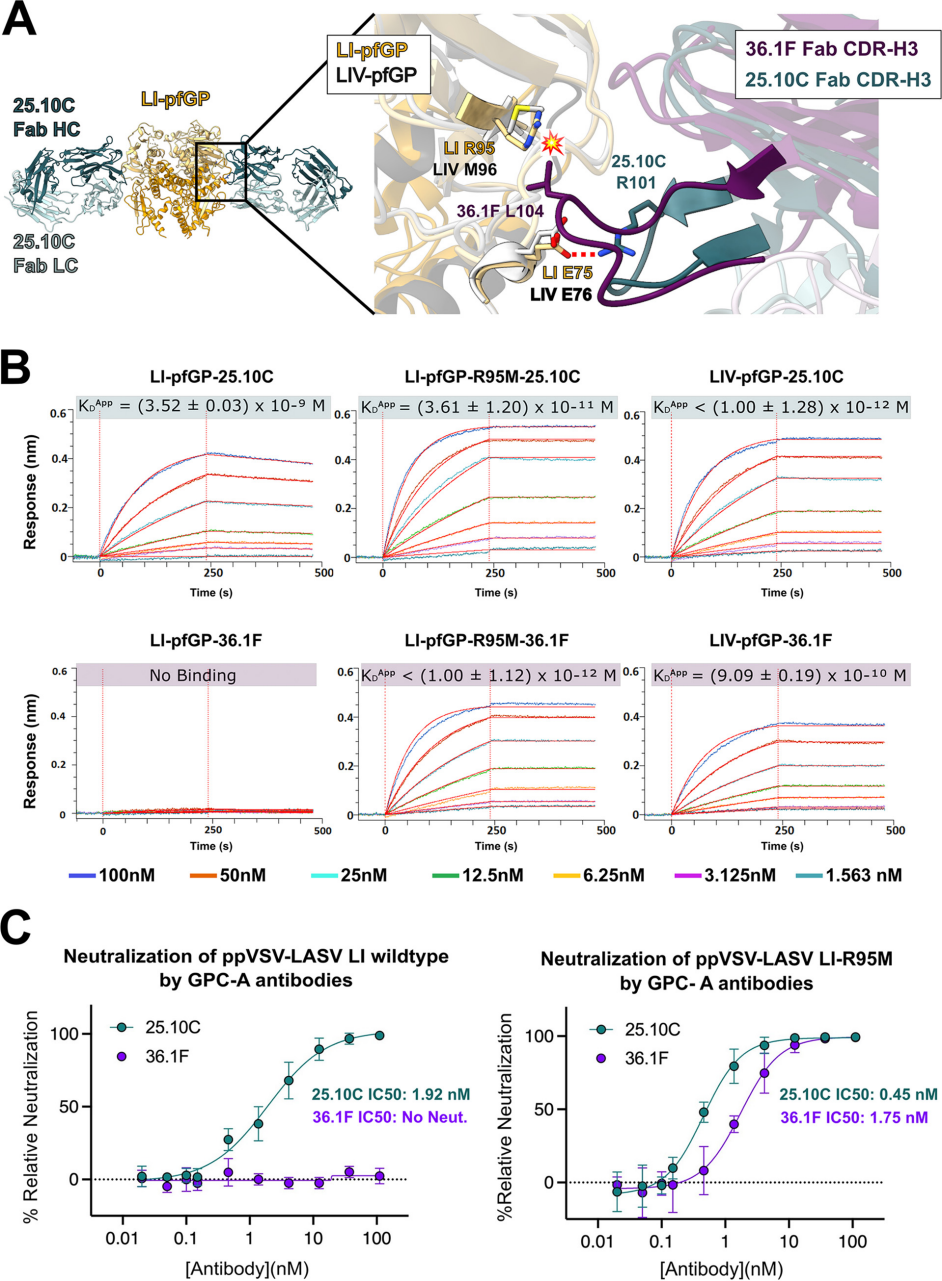
GPC-A antibody-mediated neutralization of LI LASV. (A) Superimposition of the LI-GP-25.10C atomic model (gold and green, respectively) with the LIV-GP-36.1F structure (gray and purple, respectively) (27). (Inset) The position of R95 in LI relative to the LIV-specific 36.1F suggests that this substitution would cause a steric clash with the CDRH3 of the antibody, indicated by a flash symbol. CDRH3 of the pan-LASV 25.10C instead makes a hydrogen bond with the conserved E75 side chain (red dots). (B) Kinetic binding curves for interaction between the GPC-A antibodies 25.10C and 36.1F with the indicated LASV GP monomer. For each panel, the raw data is colored according to GP concentration with the 1:1 fit shown in red. Each experiment was repeated twice, producing similar trends; results from one experiment are shown. (C) Neutralization of wild-type ppVSV-LI-GP and ppVSV-LI-GP bearing an R95M substitution by GPC-A MAbs 25.10C (green) and 36.1F (purple). Each data point is the average of two biological replicates, where each replicate was performed in technical duplicate, with the error bars indicating the standard deviation (SD) from the mean. The data were normalized to infection of Vero cells by ppVSV-LI-GP without MAb.

Of the two known anti-LASV GPC-A MAbs, only 25.10C is effective against LI. The other, 36.1F, is LIV-specific and does not neutralize LI (26). The lineage-specific GPC-A MAb 36.1F binds an epitope that is highly conserved between LI and LIV (27). Indeed, position 95 is the only nonconserved residue at the 36.1F binding site between the two lineages (see Fig. S4 in the supplemental material). Moreover, the loop containing R95 exhibits a similar conformation between LI and LIV GP (Fig. 2A). Thus, a Met or Arg residue at position 95, rather than any conformational alteration, likely determines antibody reactivity at that site. Modeling suggests that the LI M95R substitution would produce a steric clash with L104 of the 36.1F CDR H3 (Fig. 2A). In contrast, the pan-LASV 25.10C has a shorter CDR H3 that does not contact residue 95 and instead interacts with E75 in GP1.

10.1128/mbio.01278-22.2 FIG S2 Data processing and validation of LI-pfGP-18.5C-M30. (A) Representative micrograph displaying LI-pfGP-18.5C-M30 Fab complexes. Scale bar, 20 nm. (B) Two-dimensional cryo-EM class averages illustrating complete occupancy of trimeric LI-GP by the engineered 18.5C-M30 Fab (maroon). (C) Fourier shell correlation (FSC) depicting the global resolution of the final LI-pfGP-18.5C-M30 Fab cryo-EM reconstruction with 3-fold rotational symmetry applied. (D) Fourier shell correlation (FSC) between the sharpened cryo-EM reconstruction and atomic model. (E) Viewing direction distribution histogram depicting the diversity of particle projection views present in the data set. (F) Three-dimensional representation of the viewing direction distribution of the particle projections used for the final cryo-EM reconstruction. Download FIG S2, TIF file, 2.8 MB. Copyright © 2022 Buck et al. 2022 Buck et al. https://creativecommons.org/licenses/by/4.0/ This content is distributed under the terms of the Creative Commons Attribution 4.0 International license.

10.1128/mbio.01278-22.3 FIG S3 Density maps with fitted atomic models of LI-pfGP-25.10C and LI-pfGP-18.5C-M30 structures. (A) Cryo-EM density map (mesh representation) for the LI-pfGP histidine triad when bound to GPC-A MAb 25.10C (A) or GPC-B MAb LI-pfGP-18.5C-M30 (B). (C) Cryo-EM density map (mesh representation) for the LI-pfGP GP2 region bound by MAb LI-pfGP-18.5C-M30. Download FIG S3, TIF file, 2.7 MB. Copyright © 2022 Buck et al. 2022 Buck et al. https://creativecommons.org/licenses/by/4.0/ This content is distributed under the terms of the Creative Commons Attribution 4.0 International license.

10.1128/mbio.01278-22.4 FIG S4 Sequence alignments of the glycoprotein precursor (GPC) for the seven currently recognized LASV lineages. The following lineages are shown: lineage I (Pinneo), lineage II (LASV-237-Nig2010), lineage III (Nig08-A18), lineage IV (Josiah strain), lineage V (Soromba-R), lineage VI (KAK-428), and lineage VII (TGO/2016/812939). All sequences were aligned to the sequence of lineage I (Pinneo) GP using Clustal Omega in Jalview 2.11.1.4 (49) with manual notations. Dots indicate consensus residues with lineage I GP. Residues that differ from lineage I are shown as text. Key residues discussed in the main text are highlighted gold. The epitopes for 25.10C and 18.5C-M30 are highlighted in green and maroon, respectively. Download FIG S4, TIF file, 0.7 MB. Copyright © 2022 Buck et al. 2022 Buck et al. https://creativecommons.org/licenses/by/4.0/ This content is distributed under the terms of the Creative Commons Attribution 4.0 International license.

To better understand how R95 affects antibody efficacy, we used biolayer interferometry (BLI) to characterize the binding kinetics of GPC-A MAbs to LI-pfGP and LI-pfGP bearing an R95M substitution. Monomeric GP was used for these studies to enable use of the 1:1 fit model during analysis. Pan-LASV 25.10C binds wild-type LI, LI-R95M, and LIV with high affinity (Fig. 2B; see also Table S3 in the supplemental material). Lineage IV-specific 36.1F does not bind wild-type LI but does bind LI, bearing an R95M mutation with kinetics and affinity comparable to those of LIV (Fig. 2B; see also Table S3). Furthermore, while 36.1F does not neutralize pseudovirus particles bearing LI (ppVSV-LI), it robustly neutralizes ppVSV-LI-R95M (Fig. 2C). These results demonstrate that an Arg residue at position 95, which occurs in the GPC-A site of lineages I, V, VI, and VII, directly inhibits MAb 36.1F binding and neutralization.

10.1128/mbio.01278-22.10 TABLE S3 BLI association and dissociation constants for interaction of LASV pfGP with the indicated antibody. Download Table S3, DOCX file, 0.02 MB. Copyright © 2022 Buck et al. 2022 Buck et al. https://creativecommons.org/licenses/by/4.0/ This content is distributed under the terms of the Creative Commons Attribution 4.0 International license.

### LI evades neutralization by most GPC-B antibodies.

The GPC-B site is a major neutralization determinant; more than half the anti-LASV antibodies target the GPC-B epitope (26). Notably, nearly all GPC-B MAbs are derived from the same heavy-chain germ line (IGHV3-21) and thus exhibit nearly identical heavy chains. Likewise, these antibodies poorly neutralize lineage I (see Fig. S5 in the supplemental material). We previously demonstrated that antibodies 18.5C, 25.6A, and 37.7H incompletely neutralize LASV-LI ppVSV pseudovirions and fail to neutralize authentic LI LASV (28, 29). BLI analysis further reveals that wild-type GPC-B antibodies exhibit more rapid dissociation from monomeric LI relative to LIV, suggesting that a high antibody off-rate may decrease antibody efficacy (see Fig. S6 and Table S3 in the supplemental material).

10.1128/mbio.01278-22.5 FIG S5 Neutralization of ppVSV-LI-GP by wild-type GPC-B MAbs. All GPC-B MAbs shown contain similar heavy chains derived from the heavy-chain germline IGHV3-21, and each poorly neutralizes ppVSV-LI. Data points are the average of two biological replicates, where each replicate was performed in technical duplicate, with error bars indicating the SD from the mean. The data were normalized to infection of Vero cells by ppVSV-LI-GP without MAb. Download FIG S5, TIF file, 0.6 MB. Copyright © 2022 Buck et al. 2022 Buck et al. https://creativecommons.org/licenses/by/4.0/ This content is distributed under the terms of the Creative Commons Attribution 4.0 International license.

10.1128/mbio.01278-22.6 FIG S6 Analysis GPC-B antibody binding kinetics to wild-type and mutant LI LASV GP monomer. Biolayer interferometry (BLI) kinetic binding curves for interaction between the indicated GPC-B antibody and monomeric LASV GP. For all samples, IgG was loaded onto huFC-AHC capture sensors that were then dipped in solutions of the GP analyte at the indicated concentrations. For each panel, the raw data is colored according to GP concentration with the 1:1 fit shown in red. Each experiment was repeated twice, producing similar trends; results from one experiment are shown. Download FIG S6, TIF file, 0.8 MB. Copyright © 2022 Buck et al. 2022 Buck et al. https://creativecommons.org/licenses/by/4.0/ This content is distributed under the terms of the Creative Commons Attribution 4.0 International license.

**FIG 3:**
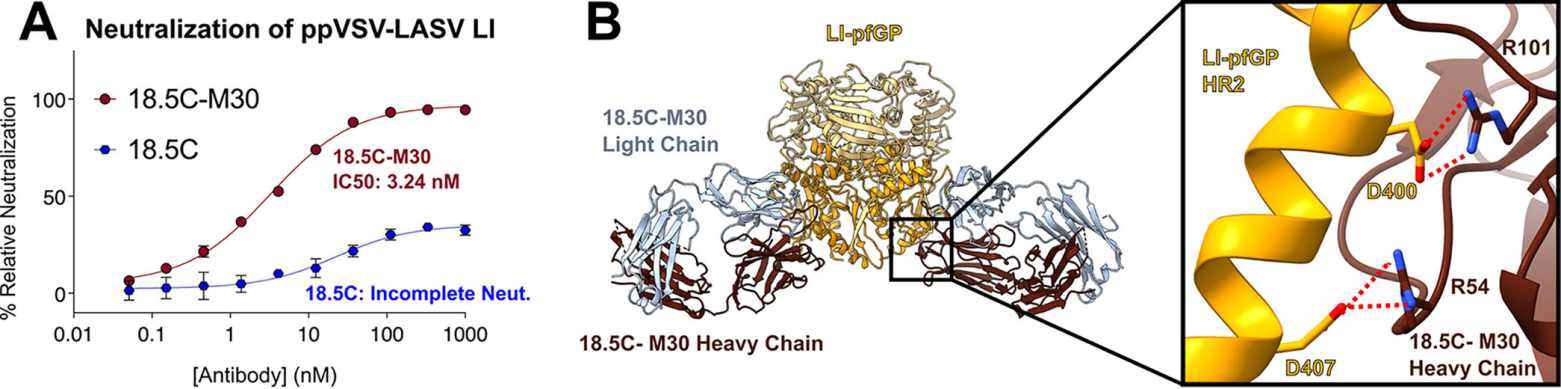
Arginine insertions increase neutralization potency of GPC-B MAb 18.5C. (A) Neutralization of ppVSV-LI by wild-type 18.5C and enhanced 18.5C-M30. Each data point is the average of two biological replicates, where each replicate was performed in technical duplicate, with the error bars indicating the SD from the mean. The data were normalized to infection of Vero cells by ppVSV-LI-GP without MAb. (B) Depictions of the novel salt-bridges (red dots) formed by engineered arginine residues at positions 54 and 101 of the 18.5C-M30 heavy chain with LI (gold) residues D400 and D407, respectively.

The GPC-B antibody 18.5C can be engineered to completely neutralize LI (Fig. 3A) (28, 29). This enhanced MAb, 18.5C-M30, introduces two Arg residues into the heavy chain via the insertion of one Arg into CDR H2 at position 54 and an Arg substitution into CDR H3 residue 100 (L100R). Here, we determined the structure of 18.5C-M30 in complex with LI-pfGP. This structure shows that the two engineered Arg residues form salt bridges to Asp400 and Asp407 of LI-pfGP (Fig. 3B). These Asp residues are conserved across all LASV lineages. Binding kinetics show that 18.5C-M30 has an increased affinity for the LI GP monomer compared to parental 18.5C, primarily due to a slower off-rate (see Fig. S6 and Table S3). Hence, neutralization of LI achieved by engineering 18.5C-M30 was likely associated with enhanced affinity to conserved parts of LASV GP.

**FIG 4:**
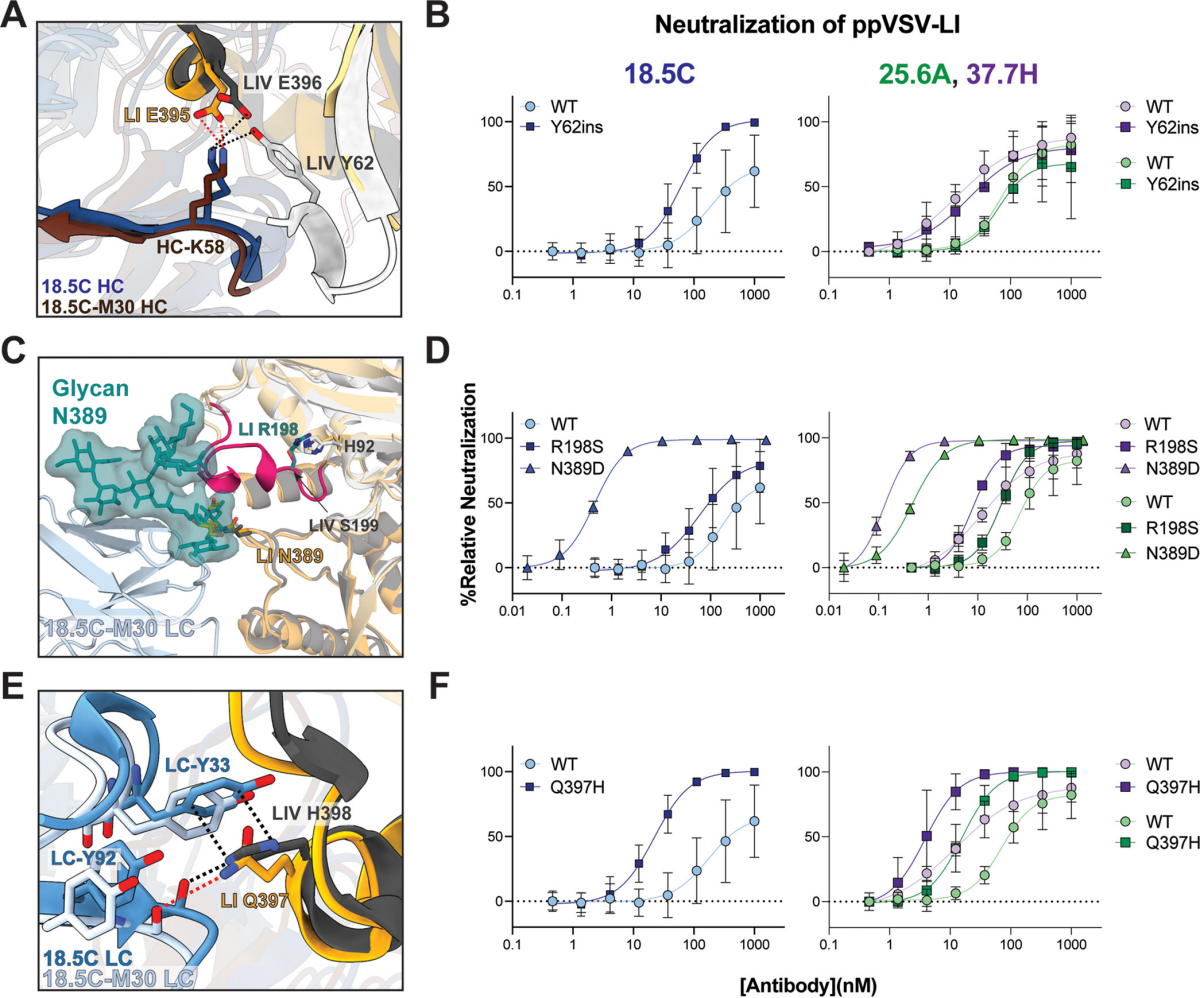
Single residue substitutions in LASV-LI decrease GPC-B antibody neutralization activity. (A) Cryo-EM structure of LI (gold) in complex with 18.5C-M30 (maroon) and crystal structure of LIV (gray) in complex with 18.5C (blue) (PDB accession number 6P91) shown as cartoons. In LIV Y62 hydrogen bonds with 18.5C heavy chain K58 (black dots) to stabilize the N-terminal β-strand of LIV. In LI, which lacks Y62, this region is disordered. (B) Neutralization of wild-type and mutant ppVSV-LI-GP bearing a Y62ins by 18.5C (left) and 25.6A or 37.7H (right). (C) Superimposition of LASV-LI GP (gold) in complex with 18.5C-M30 (light blue) and LASV-LIV GP (gray) shown as a cartoon. The 196 to 207 loop is colored magenta with residue S199 shown as sticks. The 196 to 207 loop is disordered in LI and likely adopts a different orientation relative to LIV, due to the S198R substitution (modeled here in cyan) that would sterically clash with surrounding GP1 elements. The FA2B glycan at position N390 (48) is modeled and oriented to the location of the single ordered NAG residue in LI GP. (D) Neutralization of wild-type and mutant ppVSV-LI-GP with either R198S or with genetic removal of glycan N389 via an N389D mutation by 18.5C (left) and 25.6A or 37.7H (right). (E) Cartoons of LI-GP and LIV in complex with 18.5C-M30 or parental 18.5C, respectively, are shown. H398 in LIV GP forms a pi-cation stacking interaction with Y33 of CRDL1 and hydrogen bonds with the mainchain oxygen of Y92 in CDRL3 (black dots). Q397 of LI can form a hydrogen bond only with the mainchain oxygen of Y92 (red dots). (F) Neutralization of wild-type and mutant ppVSV-LI-GP bearing a Q397H mutation by 18.5C (left) and 25.6A or 37.7H (right). In panels B, D, and F, each data point represents the average of two biological replicates, each performed in technical duplicate, with error bars indicating the SD from the mean. Data were normalized to infection by ppVSV-LI-GP without MAb. The same wild-type ppVSV-LI neutralization data are shown in each panel for comparison.

Our LI-18.5C-M30 structure pinpoints the following three LI mutations at or near the GPC-B site that decrease antibody efficacy and, thus, are responsible for the immune evasion by LI: a deletion of position Y62 and substitutions at positions 198 and 397 (Fig. 4). Most LASV lineages contain a Tyr at position 62, while LI has a deletion at this position. In LIV, Y62 hydrogen bonds with 18.5C K58 in the heavy-chain CDR H2 (Fig. 4A) (28, 29). Pseudovirions bearing wild-type LI GP are poorly neutralized by parental 18.5C, but those bearing LI with a LIV-like Y62 insertion achieve complete neutralization (Fig. 4B, left), indicating that Y62 is a critical determinant of neutralization for 18.5C. Other GPC-B MAbs like 25.6A and 37.7H are different: introduction of Y62 into LI GP does not improve neutralization by either (Fig. 4B, right). Both 25.6A and 37.7H have a His instead of Lys at residue 58, and neither forms a hydrogen bond with residue Y62 in LIV GP (28, 29).

LI also contains an Arg substitution at position 198 instead of the Ser found in all other lineages. This substitution is located in a helix spanning residues 196 to 207 that lies just above the GPC-B epitope and is adjacent to a complex glycan at N389 that sterically occludes binding by GPC-B antibodies (Fig. 4C) (28, 29). In X-ray crystal structures of LIV GP, the 196 to 207 helix is ordered, and the Ser 198 side chain faces the interior of GP (28, 29). In both LI cryo-EM structures presented here, this loop is disordered (residues 196 to 207). Modeling suggests that the R198 substitution may shift this helix outward to create space for the bulky Arg sidechain, which in turn may shift the N389 glycan even further over the GPC-B epitope, increasing the steric hindrance in this region (Fig. 4C). Indeed, removal of this glycan via an N389D mutation results in complete neutralization by 18.5C, 25.6A, and 37.7H (Fig. 4D). Furthermore, the R198S mutation improved neutralization of LI pseudovirions by 18.5C, and particularly improved neutralization by the two other GPC-B antibodies 25.6A and 37.7H. Kinetic analysis of GPC-B MAb binding to monomeric LI GP bearing the R198S mutation shows little difference in on- or off-rate compared to wild-type LI GP (Fig. S6 and Table S3). Hence, the increased neutralization afforded by this mutation is likely due to increased access to the GPC-B epitope rather than through an increase in antibody affinity for GP.

The third substitution at the GPC-B site is at LI Gln 397 (His 398 in LIV). In LIV, His 398 contacts the light chain of GPC-B MAbs 18.5C, 25.6A, and 37.7H, including a critical pi-stacking interaction with 18.5C-LC Y33 Fig. 4E (28, 29). In LI GP, however, Gln 397 cannot form stacking interactions with the 18.5C-M30 light chain, and by extension, likely fails to make the analogous contacts with 25.6A and 37.7H (Fig. 4E). Mutating LI Gln 397 to the LIV histidine considerably increases both neutralization potency and restores binding affinity to monomeric GP across all GPC-B MAbs (Fig. 4F; see also Fig. S6 and Table S3).

Together, these three LI mutations at positions 62, 198, and 397 contribute to the immune evasion of LI from GPC-B antibodies. The impact of positions 62 and 198 varies by antibody, but Gln 397 markedly impacts every GPC-B MAb tested here by decreasing both affinity and neutralization potency. Thus, Gln 397 is likely the most impactful substitution related to antibody-mediated neutralization of LI at the GPC-B site.

### LI LASV exhibits a greater dependency on LAMP1 for viral entry.

**FIG 5:**
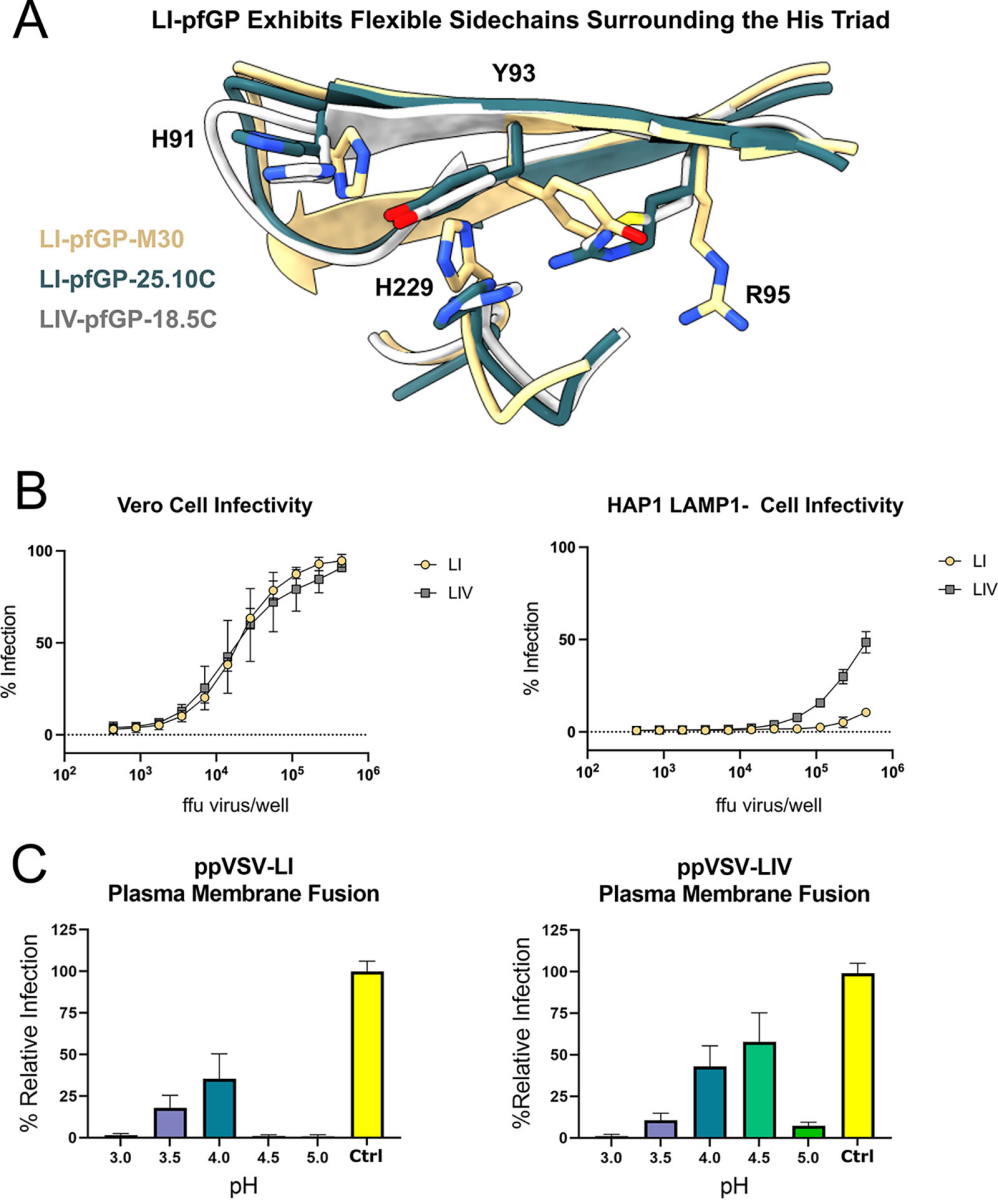
Greater dependency on LAMP1 for cellular entry of LI-GP relative to LIV-GP. (A) Superimposition of LI with 18.5C-M30, LI with 25.10C, and LIV with 18.5C (PDB accession number 6P91) at the histidine triad. Residues R95, Y93, and H229 exhibit greater mobility in LI-GP than LIV-GP. (B) Infectivity assays measuring relative infection of Vero cells and HAP1/LAMP1^−^ cells by pseudovirus as a function of virus concentration. ppVSV-LI-GP and ppVSV-LIV-GP are shown in gold and gray, respectively. Each data point represents the mean percentage of cells infected with pseudovirus. Error bars indicate the SD from the mean of two biological replicates that each have two technical duplicates. (C) Fusogenic profile of ppVSV-LASV in Vero cells as a function of pH. Each data point is the average of two biological experiments, each with four technical replicates. Error bars indicate the SD from the mean. Data were normalized to the maximum infectivity of ppVSV-LI-GP as 100% of the control in which pseudovirions naturally infected cells in the absence of treatment with acid or lysosomotropic agents (NH_4_Cl).

Thus far, interactions of Lassa virus with the LAMP1 receptor have been characterized for LIV-GP only (20, 22, 23). Our cryo-EM structures of LI GP revealed alternate conformations of rotamers in regions of GP implicated in LAMP1 binding, particularly the pH-sensing residues H91 and H229 and adjacent residues Y93 and R95 (Fig. 5A). In light of the importance of this region in LAMP1 binding, we analyzed the ability of pseudovirus particles bearing LI- or LIV-GP (ppVSV-LI or -LIV) to infect haploid cells in the absence of LAMP1 (HAP1/LAMP1^−^) or the presence of native endosomal expression of LAMP1. When LAMP1 is present, LI and LIV pseudovirus display equal levels of infectivity. In contrast, when LAMP1 is knocked out, infection of ppVSV-LI decreases considerably relative to ppVSV-LIV, indicating that LI-GP is more dependent on LAMP1 for efficient viral entry than LASV-LIV (Fig. 5B).

LAMP1 is not absolutely essential for cell entry; LIV LASV infects at 15 to 30% of wild-type levels in LAMP1-knockout cells (Fig. 5B) (20, 23). LAMP1 does increase efficiency of infection, however, by increasing the pH threshold at which membrane fusion occurs to a pH of ≤5.5 (20, 22, 23). To investigate why LI is more dependent on LAMP1 for cell entry, we examined the fusogenic profile of LI versus LIV in the absence of LAMP1, using acid-bypass assays that force viral membrane fusion to occur at the cell surface rather than the endosome. Here, LIV requires a pH of ≤4.5, while LI requires a pH of ≤4.0 to enable optimal cell entry (Fig. 5C). Hence, the increased dependency on LAMP1 displayed by LI relative to LIV is likely linked to the more acidic pH required for fusogenic activity. LI may require the more hydrolytic low-pH of late endosomes to fuse in a LAMP1-independent manner and, therefore, have a far lower infection efficiency.

The mechanism behind the more acidic fusion pH required for LI is currently understudied. Residue R95 is positioned near residues H91 and H229 (H92 and H230 in LIV), which belong to the LASV His triad and mediate the pre- to postfusion conformational changes required for viral entry (22). Hence, the R95 substitution may modulate the ability of these residues to sense pH and, therefore, alter interactions with LAMP1 in the endosome. Similarly, R198 is located in a loop implicated in LAMP1 binding, and mutation of residues adjacent to this position reduces interaction with recombinant LAMP1 (32). To understand if either of these variations in LI GP are responsible for its greater dependence on LAMP1, we analyzed whether R95M and R198S pseudovirions displayed differential fusogenic profiles compared to wild-type LI. Additionally, we examined pseudovirions including K55R, Y62ins, and Q397H mutations, since LI exhibits unique mutations at these sites. However, each mutation showed a similar profile to that of wild-type LI pseudovirions (see Fig. S7 in the supplemental material), suggesting the molecular basis for the more acidic fusion pH required by LI lies beyond these single point mutations.

10.1128/mbio.01278-22.7 FIG S7 Cell surface fusion assays of various point mutations in LI-GP. Fusogenic profile of ppVSV-LI-GP bearing the indicated point mutations in Vero cells. For all panels, each data point is the average of two biological experiments, each with four technical replicates. Error bars indicate the SD from the mean. Data were normalized to the maximum infectivity of ppVSV-LI-GP as 100% of the PBS control in which pseudovirions naturally infected cells in the absence of treatment with acid or lysosomotropic agents (NH_4_Cl). Total fusion varied among the LI-GP mutants, but wild-type LI-GP and all mutants promoted fusion at a pH of 3.5 to 4.0. Download FIG S7, TIF file, 0.2 MB. Copyright © 2022 Buck et al. 2022 Buck et al. https://creativecommons.org/licenses/by/4.0/ This content is distributed under the terms of the Creative Commons Attribution 4.0 International license.

## DISCUSSION

The genetic diversity exhibited between LASV lineages and the propensity for these lineages to evolve are major hurdles toward the design of broadly reactive therapeutics and vaccines (31). LI is the most genetically divergent lineage of LASV relative to the prototypical LIV (33). This work emphasizes the impact of single residue substitutions on antibody efficacy and further explains why the majority of neutralizing antibodies against the immunodominant GPC-A and GPC-B epitopes on LASV GP are ineffective against LI.

At the GPC-A site, the LI R95 substitution alone prevents neutralization by the antibody 36.1F. R95 is also observed in lineages V, VI, and VII and likely inhibits 36.1F-mediated neutralization of these lineages as well. Likewise, LASV lineages II and III contain nearby mutations at GP1 residues 73 and 75. These lineages, like LI, are also refractory to neutralization by 36.1F. Hence, this region may be a hot spot for viral evolution and highly vulnerable to escape by antibodies that bind in a 36.1F-like manner. Inclusion of R95 in future vaccine candidates may help elicit antibodies that neutralize in a 25.10C-like manner.

At the GPC-B site, neutralization is dictated by a network of contacts between antibody and GP as well as access to the site itself. For 18.5C, engineering novel GP contacts through an R54 insertion and an L100R mutation in the heavy chain can rescue its ability to completely neutralize LI. Curiously, GPC-B MAbs 25.6A and 37.7H have naturally occurring arginine residues in their heavy chain CDRs (R31 and R100 in 25.6A and R55 and R100 in 37.7H), yet neither of these antibodies can neutralize wild-type LI. We also find that each of the GPC-B antibodies examined here show improved neutralization of LI GP bearing an R198S mutation, which we hypothesize increases access to the GPC-B epitope. Similarly, removal of the N389 glycan that occludes the GPC-B site also enables robust neutralization by GPC-B antibodies. Hence, site access is likely an additional determinant for GPC-B efficacy against the divergent LASV LI.

Wild-type 18.5C poorly neutralizes ppVSV-LI but completely neutralizes LI with point mutations that allow for new hydrogen bonds via the insertion of Y62 in GP1 or the Q397H substitution in GP2. Moreover, the substitution of LI Q397 to the His found in all other lineages also enables neutralization by 25.6A and 37.7H—making it the only GPC-B mutation to equally impact all three GPC-B MAbs examined in this study. This residue is centrally located in the GPC-B site, and the identity of this residue appears to be a critical determinant for this class of antibodies. At the GPC-B site, specifically including Q397 in future vaccine candidates may help elicit a broadly reactive immune response by forcing novel GPC-B MAbs to abandon the pi-stacking interaction with LIV H398 and generate 18.5C-M30-like antibodies.

Furthermore, we found that LI has a greater dependence on LAMP1 for efficient entry than LIV. When LAMP1 is knocked out, ppVSV-LI infectivity is considerably less efficient than infection by ppVSV-LIV. The underlying molecular basis for this phenomenon lies beyond single substitutions in LI. Certainly, these results suggest variability in receptor dependence among LASV isolates and merit further exploration within and beyond LI.

Promising vaccine platforms that present full-length LASV GP have focused solely on LIV (34–36). Our data suggests that antibodies elicited from vaccination with LIV-GP may lack sufficient efficacy against LI and LI-like viruses, such as the newly emergent lineages LV to LVII, which each bear the same M95R mutation as LI. Mutations at the GPC-B site may be particularly important, as they render LI resistant to the most immunodominant group of anti-LASV neutralizing antibodies. Future vaccine candidates may, therefore, benefit from an immunogen that incorporates the LI-like R95 and/or Q397 to promote the formation of pan-LASV antibody interactions that may better accommodate variations at these positions. Moreover, to avoid the undesirable consequence of eliciting antibodies that do not target LIV M95/H397, a mixed immunogen or prime/boost strategy may provide an alternative approach to elicit a more broadly reactive response than solely including LI or LIV-like glycoproteins.

Overall, the studies here highlight key determinants on LASV-GP that will promote the development of affordable, broadly-reactive, and widely-accessible medical countermeasures against the multiple LASV lineages. Moreover, a comprehensive understanding of the molecular features on LASV-GP from different lineages will narrow the prioritization of therapeutics and vaccines to test in future studies.

## MATERIALS AND METHODS

### Cell lines.

HEK293T (ATCC CRL-3216) and Vero (ATCC CCL-81) cells were cultured in high-glucose Dulbecco’s modified Eagle’s medium (DMEM) containing l-glutamine (Invitrogen, Carlsbad, CA) supplemented with 10% fetal bovine serum (FBS) (Omega Scientific, Tarzana, CA) and 1% penicillin-streptomycin solution (Penn/Strep). Cells were maintained at 37°C in a humidified atmosphere with 5% CO_2_. ExpiCHO cells were cultured in ExpiCHO expression medium and maintained at 37°C in a humidified atmosphere with 8% CO_2_. *Drosophila* S2 cells were cultured in Schneider’s *Drosophila* medium at 27°C in stationary flasks. Stable cell lines were adapted to serum-free conditions and maintained with shaking at 27°C.

### Expression of IgG.

IgGs were expressed and purified according to references 27–29. Briefly, ExpiCHO-S cells were grown in shaker flasks in ExpiCHO expression medium in a humidified chamber at 37°C and 8% CO_2_. Cells were passaged every 3 to 4 days during early-log-phase growth at 4 × 10^6^ to 6 × 10^6^ cells/mL. One day before transfection, cells were diluted with prewarmed ExpiCHO expression medium to a final density of 3 × 10^6^ to 4 × 10^6^ cells/mL. On the day of transfection, the cell density was adjusted with ExpiCHO expression medium to 6 × 10^6^ cells/mL. Cells were transfected with a 1 to 1.15 ratio of heavy-chain to light-chain plasmid DNA using an ExpiFectamine CHO transfection kit. Cells were fed the next day according to the manufacturer's instructions. Antibodies were purified from clarified supernatants using protein A affinity chromatography via a HiTrap PrismA MAbSelect column. IgGs were eluted with 0.1 M citrate buffer, pH 3.4, and neutralized with a 1/10 volume of 1 M sodium phosphate, pH 8.0, before dialysis into phosphate-buffered saline (PBS).

### Production and purification of Fab fragments from IgG.

Purified IgG antibodies were digested by incubating with 5% papain (wt/wt) for 3 h at 37°C. The resulting Fab fragments were then purified using a Kappa select column (GE Healthcare) and further purified by size exclusion chromatography (SEC) using an S75 Increase 10/300 column (GE Healthcare).

### Expression and purification of LI-pfGP and derivatives.

Expression and purification of soluble LI-LASV-pfGP ectodomain monomers were performed as previously described (27–29). Briefly, the LI-LASV-GP soluble ectodomain monomer (residues 1 to 424) was modified to introduce the cysteine mutations K206C and G359C, a helix breaking E328P mutation, and the mutations L257R and L258R to alter the native S1P cleavage site to a furin protease cleavage site for production in *Drosophila* S2 cells (Invitrogen). This construct also contains an added LPETG amino acid sequence at the LI-GPCysR4 C terminus that allows ligation to the trimerization domain (PDB accession number 1NOG). S2 cells were grown to a density of 1 × 10^7^ cells/mL, and protein expression was induced using 500 μM CuSO_4_. Protein was purified from the supernatant by Strep-Tactin affinity chromatography and the StrepII tags removed by overnight incubation with EKMax (Thermo Fisher). The resulting protein was further purified by SEC using an S200 Increase column (GE Healthcare).

### LI-pfGP trimerization and purification.

LI-pfGP trimer was formed according to reference 27. Briefly, 40 μM LI-pfGP monomer was ligated to 14 μM 1NOG trimerization domain using 1.35 μM Sortase A enzyme in buffer containing 50 mM Tris, pH 7.5, 150 mM NaCl, and 2 mM CaCl_2_ and incubating for 1 h at room temperature. The ligation reaction was quenched with 50 mM iodoacetamide. The resulting LI-pfGP trimer was purified using an S200 Increase column.

### Antibody-LI-pfGP complex formation.

Purified LI-pfGP trimer was incubated with excess Fab for at least 1 h at room temperature (RT). LI-pfGP trimer-Fab complexes were then purified by SEC using an S200 Increase column (GE Healthcare).

### Cryo-EM sample preparation and data collection.

Purified LI-LASV-pfGP-25.10C complexes were concentrated to 0.7 mg/mL, and a 3-μL aliquot was mixed with 1 μL 0.02 mM lauryl maltose neopentyl glycol (LMNG) detergent. No detergent was added to the LI-pfGP-18.5C-M30 complex. The samples (3 μL) were applied to C-flat 2/1 copper grids that had been plasma cleaned for 30 s in a NanoClean model 1070 (Fischione Instruments) using a mixture of 25% oxygen and 75% argon. Grids were blotted for 10 s to remove excess solution and then plunge-frozen in liquid ethane using an FEI Vitrobot (Thermo Fisher Scientific). Frozen grids containing LI-pfGP-25.10C were imaged using a Titan Krios (Thermo Fisher Scientific) equipped with a Gatan K2 detector, while grids containing LI-pfGP-18.5C-M30 were imaged using a Titan Krios equipped with a Gatan K3 detector. Movies for the LI-pfGP-25.10C complex were collected at a magnification of ×46,300 in super resolution mode and correspond to a calibrated pixel size of 0.548 Å/pixel. Movies were collected in a single session with a defocus range between 1.0 and 2.5 μm underfocus. Movies for the LI-pfGP-18.5C-M30 complex were collected at a magnification of ×75,750 in counting mode that corresponds to a calibrated pixel size of 0.6656. A full description of the cryo-EM data collection parameters is presented in Tables S1 and S2 in the supplemental material.

### Cryo-EM data processing.

All data processing was carried out using cryoSPARC v2.14.2 (37). Movies were motion-corrected using Patch motion correction and subsequently contrast transfer function (CTF) corrected using Patch CTF estimation.

For the LI-GP-25.10C complex, particle projections were picked from the micrographs using Topaz (38), downsampled by a factor of 2, and then subjected to reference-free two-dimensional (2D) class averaging to generate a particle stack of 152,874 particles that were used for *ab initio* reconstructions and subsequent three-dimensional (3D) and CTF refinements. The final C3 symmetrized reconstruction reported a resolution of 3.09 Å according to the gold standard Fourier shell correlation criterion (39).

For the LI-pfGP-18.5C-M30 sample, particle projections were also picked using Topaz (38) but were downsampled by a factor of 3 before reference-free 2D averaging, which generated a final particle stack of 380,210 particles. After generating an *ab initio* with subsequent 3D refinements, the micrographs were unbinned so that they were downsampled by a factor of 1.95 before undergoing a final series of 3D and CTF refinements. The final C3 symmetrized reconstruction reported a resolution of 3.59 Å according to the gold standard Fourier shell correlation criterion (39).

For both samples, postprocessing of the cryo-EM reconstruction was performed using DeepEMhancer (40). All cryo-EM reconstructions were visualized using Chimera (41) and ChimeraX (42) software packages. Three-dimensional viewing distributions were obtained using PYEM (43).

### Atomic model building into cryo-EM reconstructions.

The LI-pfGP-25.10C atomic model was built using the previously determined cryo-EM structure of the LIV-LASV-pfGP-25.10C complex (27). The LIV-pfGP-25.10C model was first docked into the LI-pfGP-25.10C cryo-EM reconstruction using Chimera (41). All sequence mutations between the LI and LIV amino acid sequence were manually corrected using Coot (44).

The LI-pfGP-M30 atomic model was built using the above LI-pfGP structure and existing 18.5C Fab model (PDB accession number 6P91). The LI-pfGP and 18.5C models were first docked into the LI-pfGP-M30 cryo-EM reconstruction using Chimera (41), and sequence mutations to the wild-type 18.5C Fab were manually corrected in Coot.

Both models were subjected to real-space refinement in Phenix (45). Finally, rotamer and Ramachandran outliers were manually adjusted using Coot (44). PDBcare (46) was used to validate the quality of glycan models. PyMOL and Chimera were used for visualization of final atomic models (47).

### Generation of LI-LASV-GPC plasmids and pseudoviruses.

Recombinant VSV-ΔG-GFP pseudovirions were prepared as previously described (28, 29). Additional LI-LASV-GP mutants were prepared via standard mutagenesis from the construct expressing wild-type LI-LASV-GP (28, 29). All plasmids were verified by Sanger sequencing. The ppVSV pseudovirions containing the LI-LASV-GP and derivative LI-LASV-GP mutants were generated by transfecting 293T cells with phCMV3 expressing the indicated version of LI-LASV-GP using TransIT-LTI (Mirus, Madison, WI) per the manufacturer’s instructions. At 24 h posttransfection, the medium was removed, and cells were infected with ppVSV-G pseudotyped ΔG-GFP parent virus (VSV-G*ΔG-GFP) at a multiplicity of infection (MOI) of 1 for 2 h, rocking every 15 min. Virus was removed, cells were washed two times with Opti-MEM containing 2% FBS (Opti-2), and fresh Opti-2 was added to cells. Supernatants containing ppVSV-LI-LASV were removed from cells 16 h postinfection and clarified by centrifugation. Titers were quantified as the number of fluorescent forming units (ffu/mL) using a CellInsight CX5 imager and automated enumeration of cells expressing green fluorescent protein (GFP). Aliquots of virus were flash frozen in liquid nitrogen and stored at −80°C.

### Neutralization assays.

Pretitrated amounts of ppVSV-LI-LASV (wild type or mutant) were incubated with the indicated concentrations of antibody at 37°C for 1 h before addition to confluent Vero monolayers in 96-well plates. Infection proceeded for 16 to 18 h at 37°C in 5% CO_2_ before cells were fixed in 4% paraformaldehyde and stained with 100 μg/mL Hoechst 33342. Cells were imaged using a CellInsight CX5 imager, and infection was quantitated by automated enumeration of total cells and those expressing GFP. Infection was normalized to the percent cells infected with ppVSV-LI-LASV without antibody present. Data are presented as the relative neutralization for each antibody concentration.

### Cell surface fusion assays.

The indicated ppVSV-LASV GP was added to prechilled Vero cells at a concentration of 35,000 ffu/well in Opti-MEM supplemented with 2% FBS and 1% Penn/Strep (Opti2PS). Viruses were adsorbed to the cell surface by centrifugation at 2,500 rpm for 1 h at 4°C. Cells were placed on ice, and unbound virus was removed through two washes with ice-cold PBS. PBS was replaced with prewarmed citrate buffer (ranging from pH 3.5 to 5.5; 50 mM citrate/150 mM NaCl) to induce fusion. Cells were incubated at 37°C for 5 min and then returned to ice and washed in medium containing 20 mM NH_4_Cl or medium alone for control wells. Finally, cells were incubated in fresh medium containing NH_4_Cl or medium alone at 37°C in 5% CO_2_. Cells were fixed, stained, and scored for infection at 16 h postinfection as described above.

All measurements were performed in quadruplicate, and data were normalized to the total infectivity of virus under native entry conditions (i.e., endosomal dependent and not exposed to either acid or lysosomotropic agents).

### Infectivity assays.

The indicated amount of ppVSV-LASV (LI or LIV) was applied to confluent Vero or HAP1/LAMP^−^ cell monolayers in 96-well plates. Infection proceeded for 16 to 18 h at 37°C in 5% CO_2_ before cells were fixed and stained as indicated above. Cells were fixed, stained, and scored for infection at 16 h postinfection as described above. Data are presented as the average percent total infection by viral concentration.

### Kinetic binding analysis by biolayer interferometry.

BLI analysis was conducted as previously described with minor adjustments (28, 29). Briefly, the Octet Red384 system was used to identify the binding properties of different IgGs and the indicated forms of soluble LASV GP monomer. Anti-human Fc (AHC) capture sensors were used for initial IgG loading at 2.5 μg/mL in 1× kinetics buffer (PBS supplemented with 0.1% bovine serum albumin [BSA] and 0.02% Tween 20). Binding of LASV GP to IgG-loaded sensors was performed with 2-fold serial dilutions of GP. Baseline and dissociation steps were carried out in 1× kinetics buffer. Regeneration was carried out using 1% phosphoric acid. Reference sensors (kinetics buffer only) were subtracted from each data set. Kinetic parameters (*K*_on_ and *K*_off_) and affinities (*K*_D_) were calculated from a nonlinear fit based on the 1:1 binding kinetic model of the data using the Octet Data analysis HT software version 11 (ForteBio). However, given the bivalent nature of the IgG (ligand) and bivalent nature of the dual-site GPC-B epitope on LASV GP (analyte), the association stoichiometry is more complex relative to available models. We therefore refer to *K*_D_s obtained here as the apparent *K*_D_ (*K*_D_^app^). A minimum of two replicates for each binding curve was performed. Table S3 contains the average values and calculated standard deviations for each kinetic measurement.

### Resource availability.

Requests for antibodies from reference 26 should be directed to Luis Branco (lbranco@zalgenlabs.com).

### Data availability.

The atomic models and cryo-EM maps derived from the Fab 25.10C bound structure and Fab 18.5C-M30 bound structure have been deposited at the PDB (http://rcsb.org) and in the Electron Microscopy Databank (EMDB) (http://www.emdataresource.org/) under the following accession numbers and are available at the time of publication: PDB accession number 7UDS and EM map EMD-26458 (LI-pfGP-25.10C) as well as PDB accession number 7UL7 and EM map EMD-26594 (LI-pfGP-18.5C-M30).

Any additional information required to reanalyze the data reported in this paper is available from the corresponding authors upon request.
