# Supplementary figures and images for: Neutralizing Antibodies against Lassa Virus Lineage I
Source: mBio. 2022 Jun 22;13(4):e01278-22. doi: 10.1128/mbio.01278-22 (PMC9426445; doi:10.1128/mbio.01278-22)

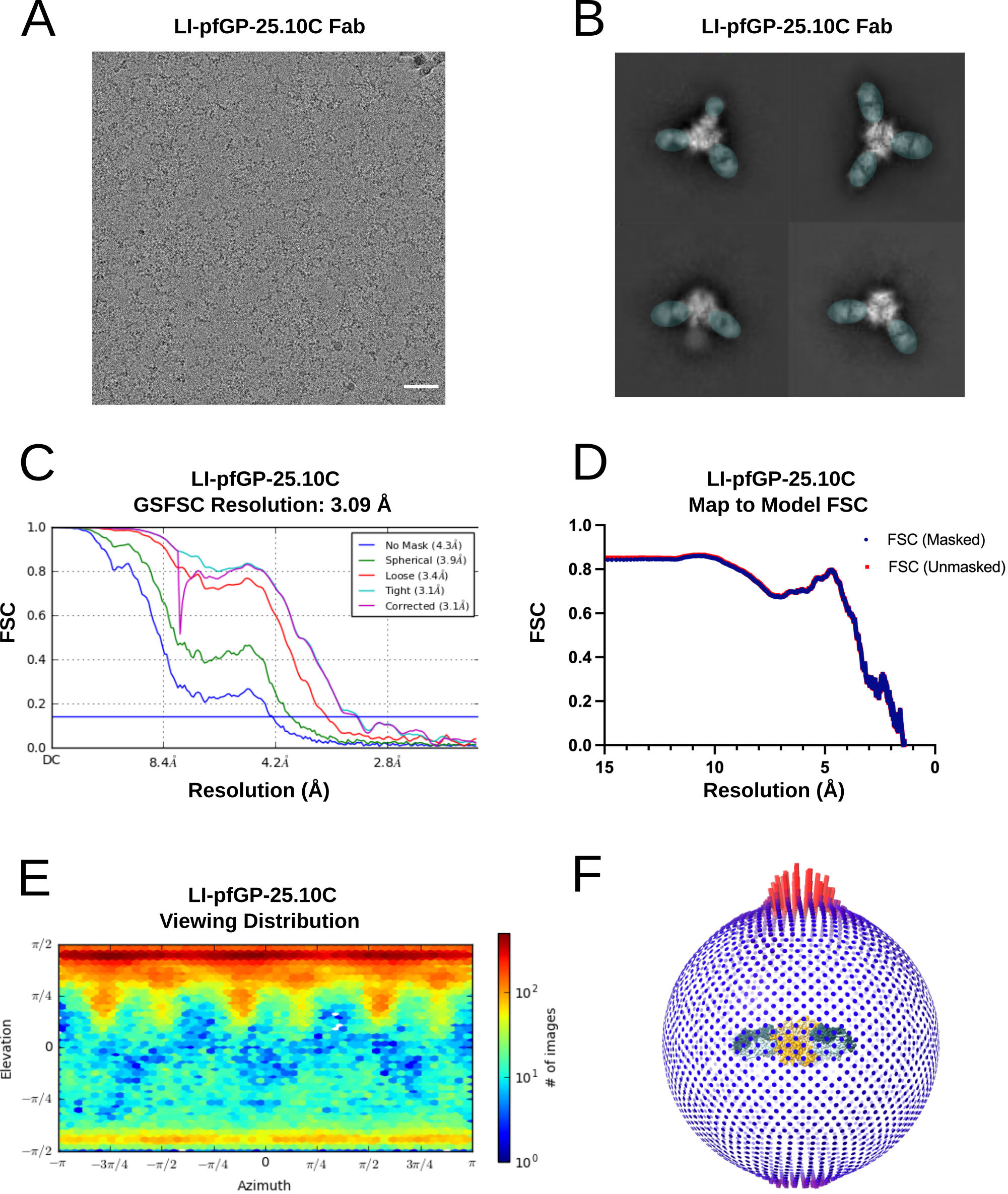

Supplement: FIG S1 [file mbio.01278-22-s0004.tif]

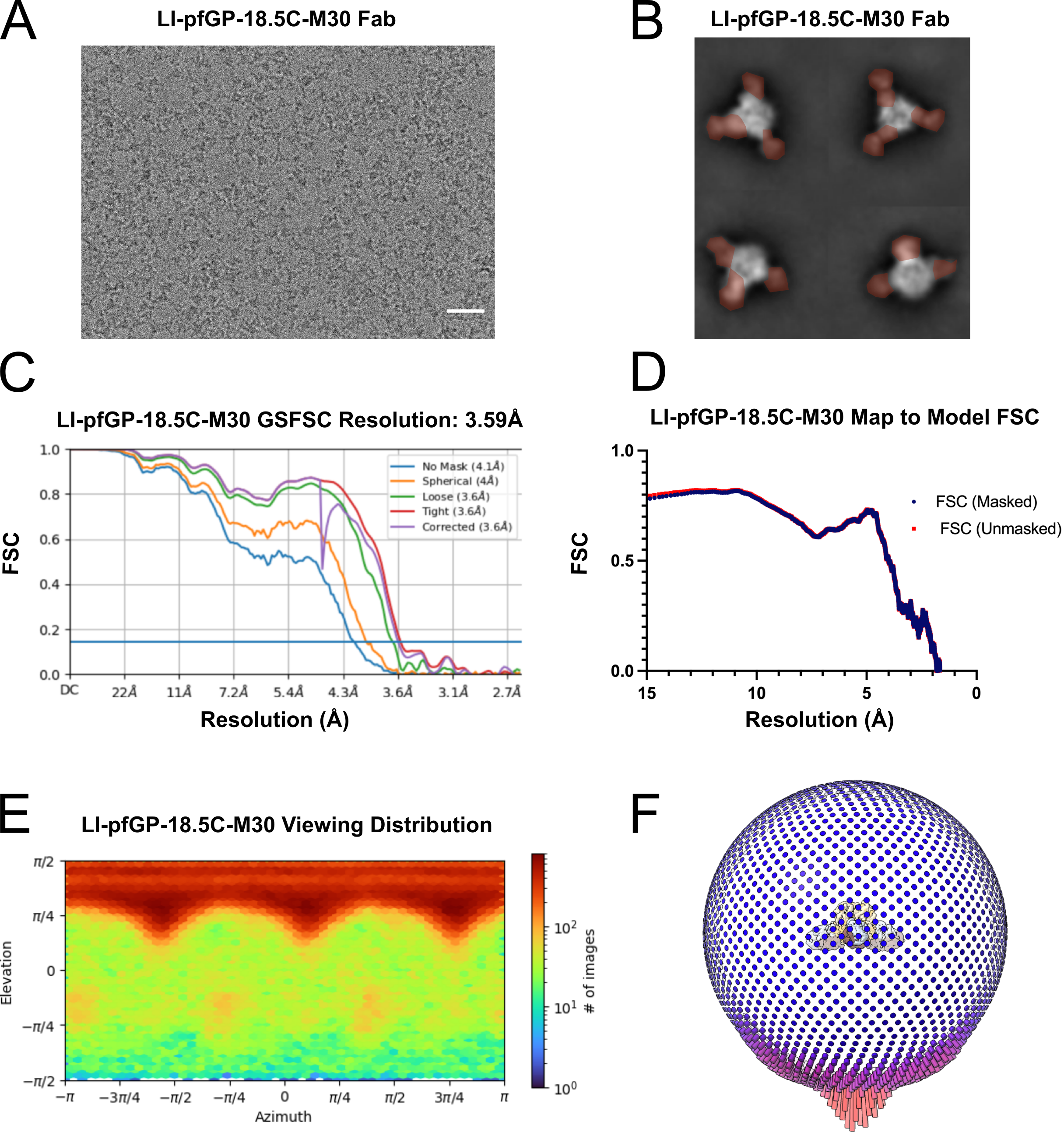

Supplement: FIG S2 [file mbio.01278-22-s0005.tif]

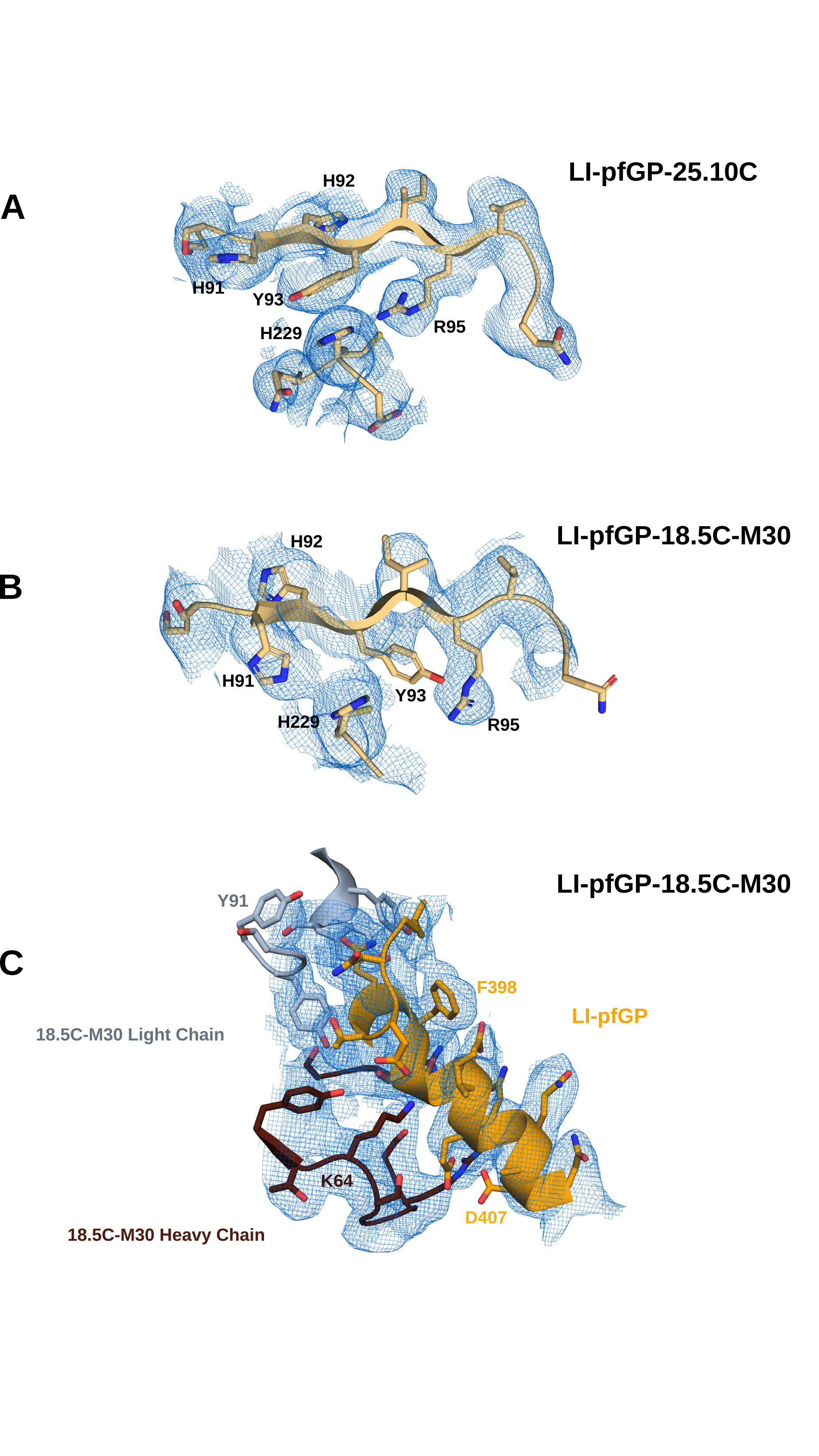

Supplement: FIG S3 [file mbio.01278-22-s0006.tif]

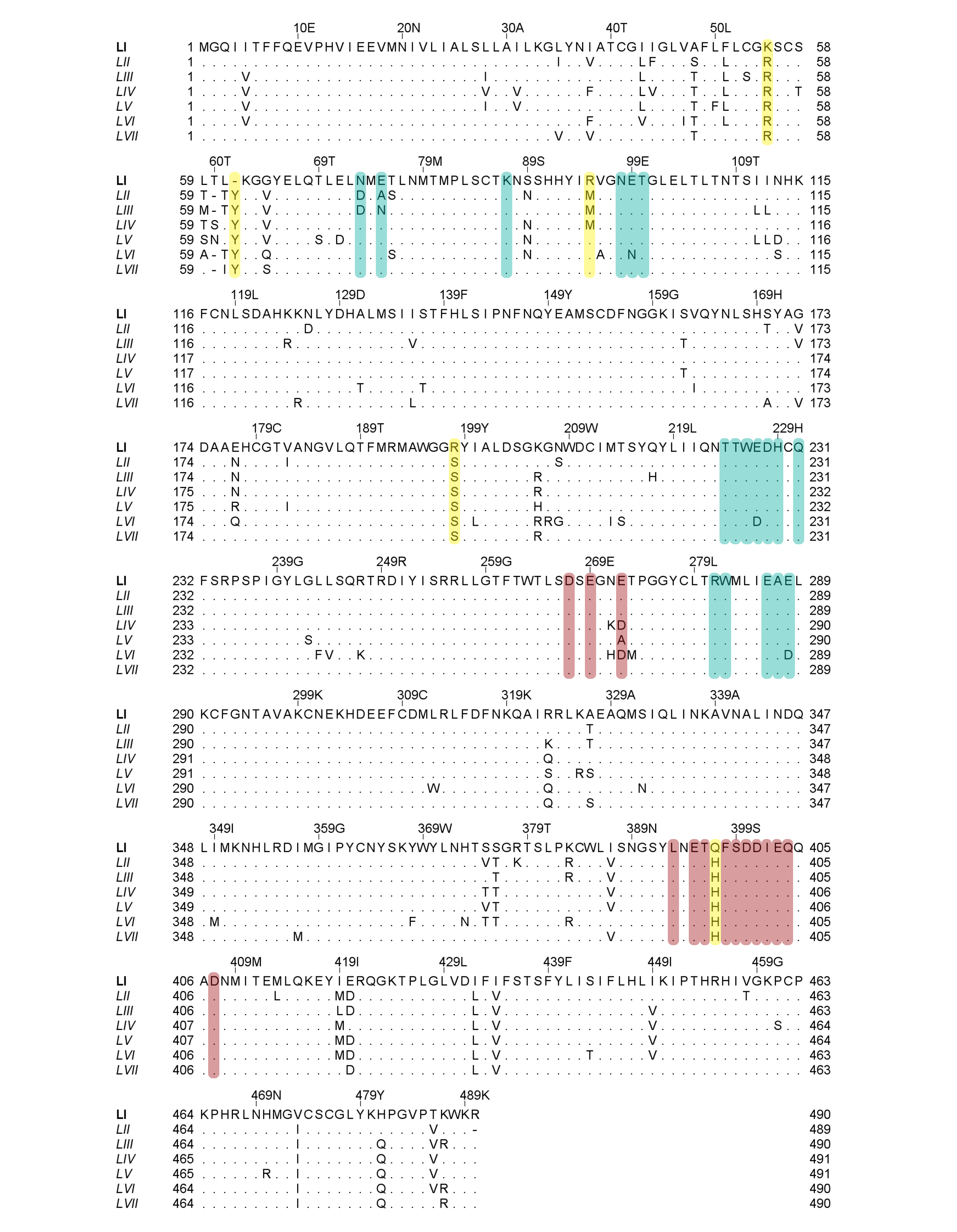

Supplement: FIG S4 [file mbio.01278-22-s0007.tif]

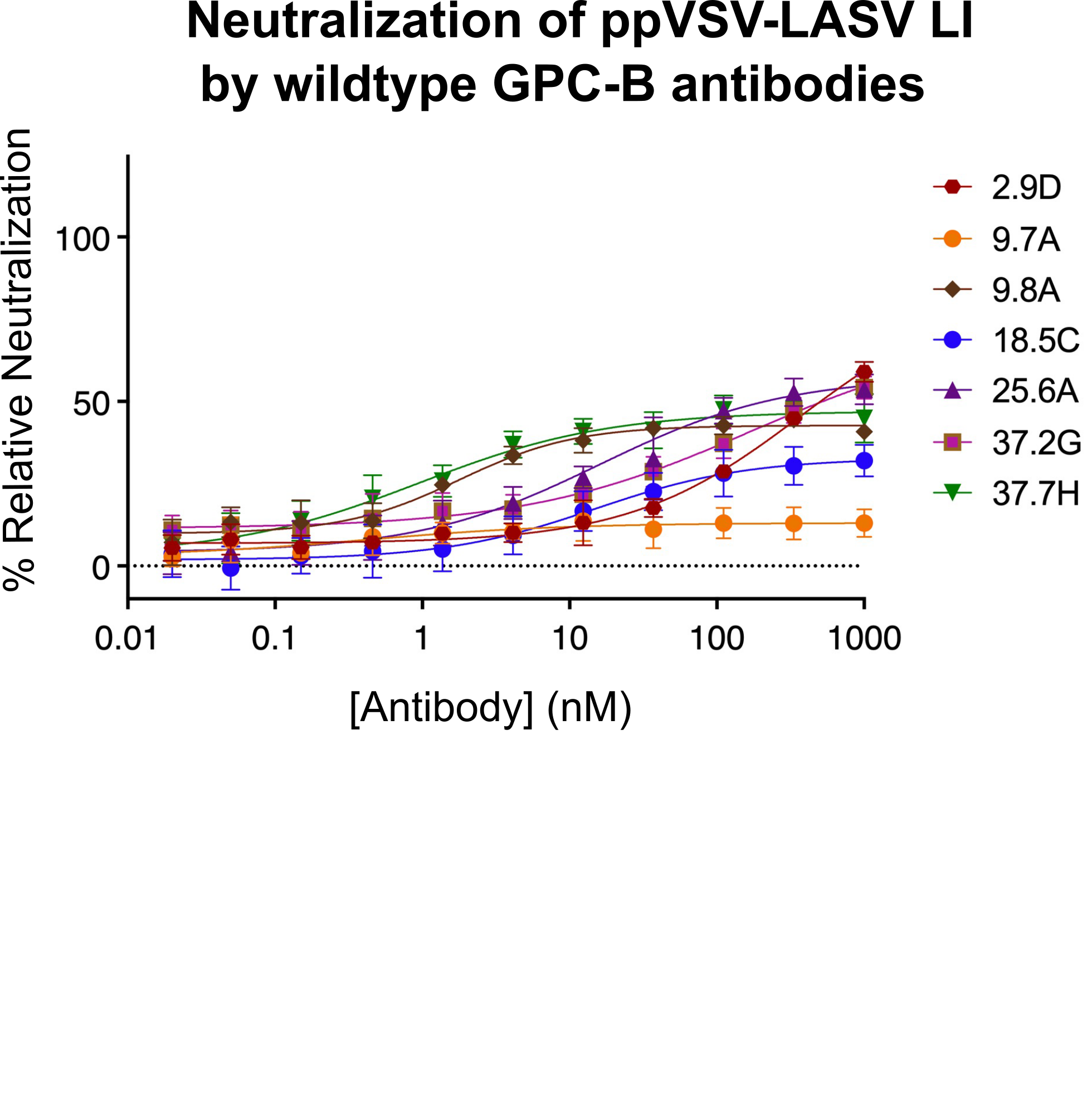

Supplement: FIG S5 [file mbio.01278-22-s0008.tif]

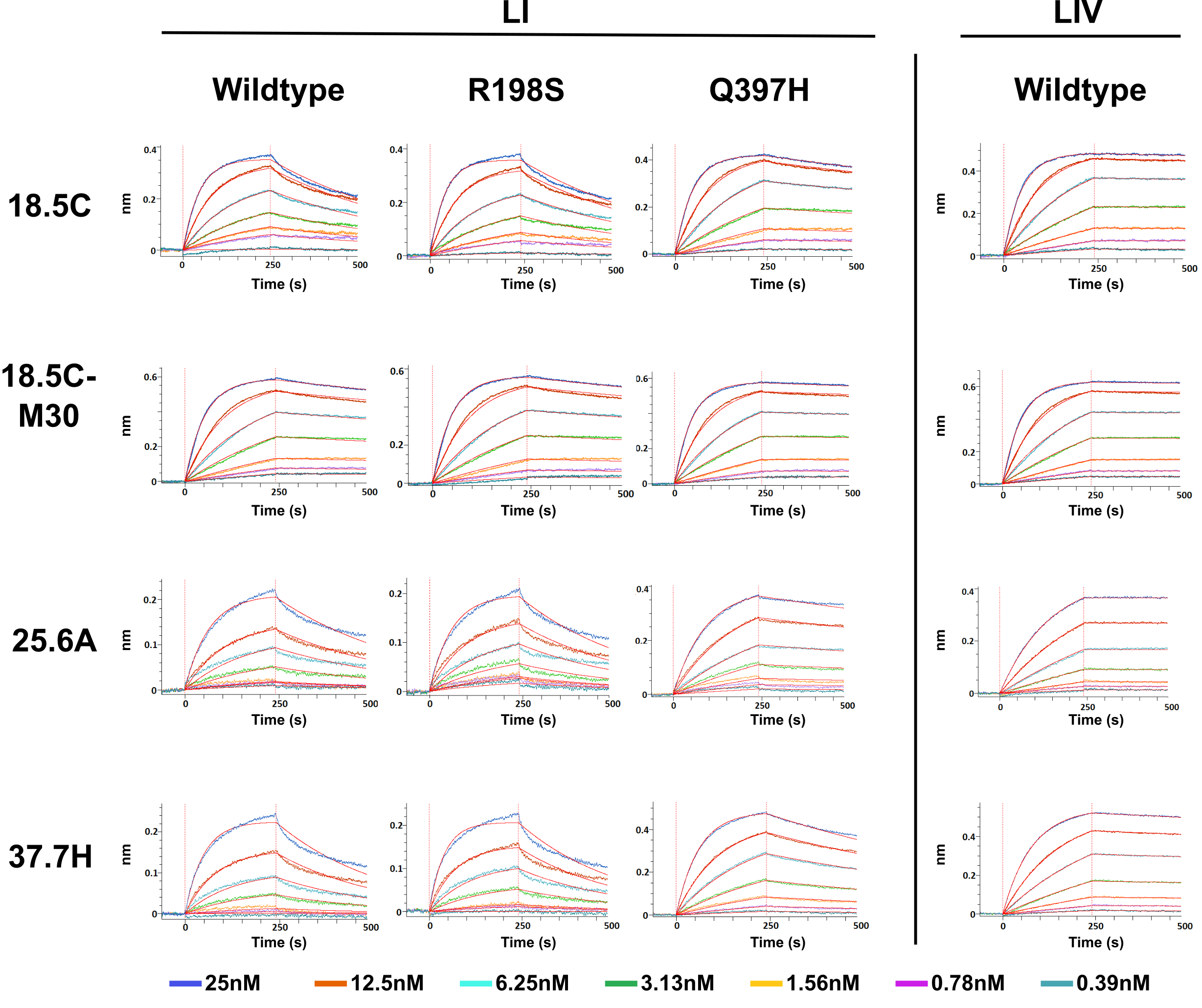

Supplement: FIG S6 [file mbio.01278-22-s0009.tif]

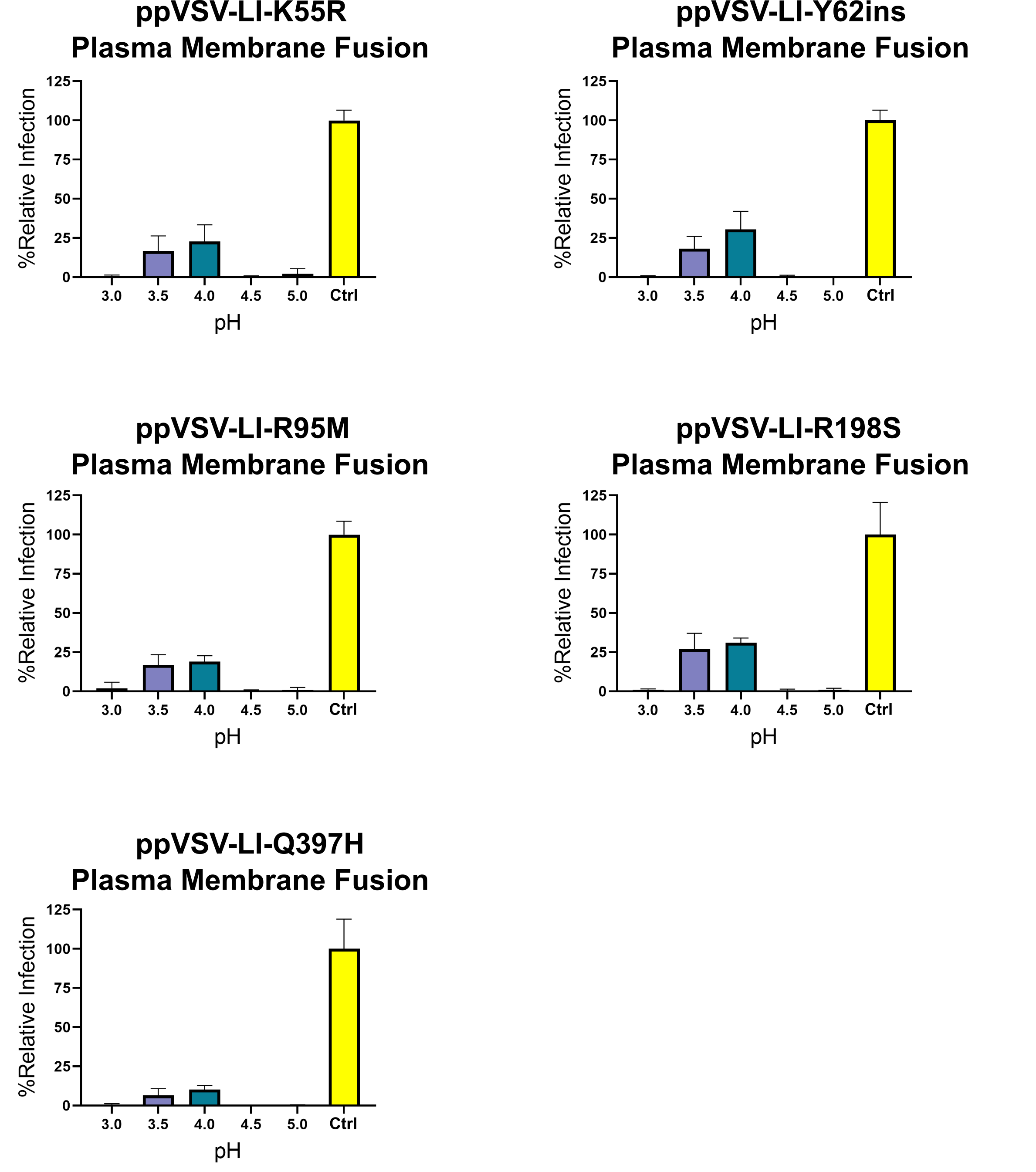

Supplement: FIG S7 [file mbio.01278-22-s0010.tif]
