# Supplementary material for: Neutralizing Antibodies against Lassa Virus Lineage I
Source: mBio. 2022 Jun 22;13(4):e01278-22. doi: 10.1128/mbio.01278-22 (PMC9426445; doi:10.1128/mbio.01278-22)
Supplement: TABLE S2 [file mbio.01278-22-s0002.docx]

**​Table S2. LI-pfGP in complex with 18.5C-M30, related to Figure 1.**

| EMDB Identifier Code  PDB Accession Code | EMDB-26594  7UL7 |
| --- | --- |
| **Data Collection** |  |
| Microscope | Titan Krios |
| Voltage (kV) | 300 |
| Detector | Gatan K3 Summit |
| Defocus range (µm) | 1.0-2.5 |
| Magnification | 75,750X |
| Movies | 4,264 |
| frames per movie | 50 |
| Exposure timer per frame (ms) | 3.6 |
| Dose rate (e-/pixel/sec) | 1 |
| Total dose per movie (e-/Å^2^) | 50 |
| Movie micrograph pixel size (Å/pixel) | 0.6656 |
| Number of particles in final reconstruction | 380,210 |
| Symmetry applied | C3 |
| Map resolution (Å) (FSC=0.143) | 3.59 |
|  |  |
| **Model Statistics** |  |
| Chains | 12 |
| Atoms | 17,439 |
| Residues (protein) | 2,178 |
| Water | 0 |
| Ligands | NAG: 30 |
| **Map to Model** | **Masked             Unmasked** |
| FSC 0/0.143/0.5 | 1.6/ 2.1/ 4.0         1.6/ 2.1/ 4.0 |
| CC* (mask) | 0.66 |
| CC (box) | 0.70 |
| CC (peaks) | 0.66 |
| CC (volume) | 0.67 |
| Mean CC for ligands | 0.66 |
| R.m.s deviations |  |
| Bond lengths (Å) | 0.004 |
| Bond angles (°) | 0.654 |
| Ramachandran plot |  |
| % favored | 93.93 |
| % allowed | 6.07 |
| % outliers | 0.00 |
| **Molprobity** |  |
| Clashscore | 13.10 |
| EM-Ringer | 2.12 |

*Cross correlation
