# Supplementary material for: Neutralizing Antibodies against Lassa Virus Lineage I
Source: mBio. 2022 Jun 22;13(4):e01278-22. doi: 10.1128/mbio.01278-22 (PMC9426445; doi:10.1128/mbio.01278-22)
Supplement: TABLE S3 [file mbio.01278-22-s0003.docx]

**Table S3**: **BLI association and dissociation constants for interaction of LASV pfGP with the indicated antibody, related to Figure 2 and Figure S6.**

| Ligand | Analyte | K_on_ (1/Ms) | K_off_ (1/s) | K_D_ ^App^ (M) |
| --- | --- | --- | --- | --- |
| LI-GP | 18.5C | (8.19 + .02) x 10^5^ | (2.35 + .005) x 10^-3^ | (2.86 + .01) x 10^-9^ |
| LI-R198S-GP | 18.5C | (9.69 + .03) x 10^5^ | (2.41 + .005) x 10^-3^ | (2.49 + .01) x 10^-9^ |
| LI-Q397H-GP | 18.5C | (8.48 + .01) x 10^5^ | (5.02 + .02) x 10^-4^ | (5.92 + .03) x 10^-10^ |
| LIV-GP | 18.5C | (8.58 + .01) x 10^5^ | (7.80 + .16) x 10^-5^ | (9.10 + .18) x 10^-11^ |
| LI-GP | 18.5C-M30 | (7.57 + .01) x 10^5^ | (4.23 + .03) x 10^-4^ | (5.59 + .04) x 10^-10^ |
| LI-R198S-GP | 18.5C-M30 | (8.13 + .02) x 10^5^ | (4.01 + .03) x 10^-4^ | (4.94 + .04) x 10^-10^ |
| LI-Q397H-GP | 18.5C-M30 | (8.37 + .01) x 10^5^ | (1.29 + .02) x 10^-4^ | (1.54 + .02) x 10^-10^ |
| LIV-GP | 18.5C-M30 | (7.80 + .01) x 10^5^ | (3.67 + .17) x 10^-5^ | (4.70 + .22) x 10^-11^ |
| LI-GP | 37.7H | (6.99 + .04) x 10^5^ | (3.53 + .01) x 10^-3^ | (5.04 + .03) x 10^-9^ |
| LI-R198S-GP | 37.7H | (8.69 + .05) x 10^5^ | (3.70 + .01) x 10^-3^ | (4.23 + .03) x 10^-9^ |
| LI-Q397H-GP | 37.7H | (6.27 + .01) x 10^5^ | (1.23 + .003) x 10^-3^ | (1.96 + .01) x 10^-9^ |
| LIV-GP | 37.7H | (5.09 + .01) x 10^5^ | (1.79 + .01) x 10^-4^ | (3.53 + .03) x 10^-10^ |
| LI-GP | 25.6A | (5.32 + .03) x 10^5^ | (2.82 + .01) x 10^-3^ | (5.30 + .03) x 10^-9^ |
| LI-R198S-GP | 25.6A | (5.70 + .04) x 10^5^ | (3.24 + .01) x 10^-3^ | (5.68 + .04) x 10^-9^ |
| LI-Q397H-GP | 25.6A | (4.28 + .02) x 10^5^ | (5.49 + .05) x 10^-4^ | (1.28 + .01) x 10^-9^ |
| LIV-GP | 25.6A | (2.79 + .01) x 10^5^ | < (1.00 + 1.47) x 10^-7^ | < (1.00 + 1.00) x 10^-12^ |
| LI-GP | 25.10C | (1.10 + .003) x 10^5^ | (3.87 + .03) x 10^-4^ | (3.52 + .03) x10^-9^ |
| LI-R95M-GP | 25.10C | (2.04 + .004) x 10^5^ | (7.36 + 2.44) x 10^-6^ | (3.61 + 1.20) x 10^-11^ |
| LIV-GP | 25.10C | (1.50 + .003) x 10^5^ | < (1.00 + 1.92) x 10^-7^ | < (1.00 + 1.28) x 10^-12^ |
| LI-GP | 36.1F | No Binding | No Binding | No Binding |
| LI-R95M-GP | 36.1F | (1.42 + .002) x 10^5^ | < (1.00 + 1.60) x 10^-7^ | < (1.00 + 1.12) x 10^-12^ |
| LIV-GP | 36.1F | (1.06 + .002) x 10^5^ | (9.63 + .20) x 10^-5^ | (9.09 + .19) x 10^-10^ |
